# Quantitative analysis of the 3D cell shape changes driving soybean germination

**DOI:** 10.1093/jxb/erx048

**Published:** 2017-04-22

**Authors:** Nátali Maidl Souza, Alexander T. Topham, George W. Bassel

**Affiliations:** 1 School of Biosciences, University of Birmingham, Birmingham B15 2TT, UK; 2 Universidade Estadual de Londrina, Programa de Pós-Graduação em Agronomia, Londrina, Brazil

**Keywords:** 3D, germination, growth, seed, segmentation, soybean.

## Abstract

Seed germination is central to plant establishment and is the starting point for the majority of world agriculture. This transition from seed to seedling has been extensively studied at an organ level, while few studies have examined the cellular events which underlie it. Reports in the model species Arabidopsis have identified a radicle-derived wave of cell expansion underlying the germination process. Whether this spatiotemporal pattern of cell expansion is specific to this model plant or conserved in other species remains unknown. Here we examined the 3D cell anisotropy driving germination in soybean. By examining changes in cell shape at two positions along the length of the axis over time, preferential growth was observed in the portion of the axis closest to the radicle. A gradient of cell size was observed across the cortical cell layers of the soybean axis, and differences in starting cell size translated into differential relative growth rates across cell layers where larger cells showed greater relative growth rates than smaller cells. Differences in cell position-specific cell anisotropy were also observed. These data demonstrate that a radicle-derived growth pattern is present in the crop species soybean, and reveal the presence of a complex cellular organization in this hypocotyl which show cell type-specific anisotropy diving germination.

## Highlight

Changes in 3D anisotropy across the soybean axis during seed germination revealed a radicle-biased pattern of cell growth and position-specific changes in cell anisotropy.

## Introduction

Seeds provide plants with the means to move through time and space (Finch-Savage and Leubner-Metzger, 2006). They are also the source of the majority of human caloric intake, and their germination represents the starting point for most global agriculture (Finch-Savage and Bassel, 2016). Following the termination of seed dormancy, the germination programme is initiated, triggering a developmentally regulated sequence of events which ultimately leads to the establishment of a plant (Bassel *et al.*, 2011; Dekkers *et al.*, 2013). The seed to seedling transition in plants is driven exclusively by cell expansion in the absence of divisions (Sliwinska *et al.*, 2009; Bassel *et al.*, 2014).

Extensive work has provided insights into the molecular regulation of seed germination at a whole-seed level (Lopez-Molina *et al.*, 2001; Koornneef *et al.*, 2002; Nakabayashi *et al.*, 2005; Cadman *et al.*, 2006; Finch-Savage and Leubner-Metzger, 2006; Bassel *et al.*, 2011; Dekkers *et al.*, 2013). However, much less is known about how these events unfold at a cellular level within the growing embryo across time.

Diverse patterns of gene expression during seed germination have been described previously using enhancer trap lines (Liu *et al.*, 2005) and through the use of targeted reporter constructs (Bassel *et al.*, 2014). Gene expression associated with cell expansion was first detected in the radicle tip of the germinating Arabidopsis embryo, and it has been proposed that the germination programme is first initiated in this subset of cells within the embryo (Bassel, 2016).

Studies have also examined the spatiotemporal pattern of cell expansion which drives the seed to seedling transition. A study examining the growth of the tomato embryo axis identified the majority of absolute growth to take place in the area adjacent to the radicle (Chen *et al.*, 2001). The enhanced growth of this region was correlated with the localized expression of the expansin gene *LeEXP8*.

More recently, studies have examined the spatial and temporal growth of cells driving Arabidopsis seed germination (Sliwinska *et al.*, 2009; Bassel *et al.*, 2014), also identifying the majority of growth to be in cells above the radicle. The examination of the early expression of growth-promoting gene expression, however, identified the cells of the radicle as the primary site of induction (Bassel *et al.*, 2014). The discrepancy between the radicle-based gene expression pattern and observed growth further down the axis was attributed to mechanical restraints imposed upon the radicle by the smaller cell sizes and tight packing of cells within this region of the embryo axis (Bassel *et al.*, 2014), explaining the difference between gene expression and observed growth.

Following the initial induction of growth-promoting gene expression in the radicle, the domain of expression extends along the length of the axis in a wave (Bassel *et al.*, 2014). This precedes the wave of growth which is observed during the germination process itself. This is also temporally distinct from the wave of growth observed during hypocotyl growth during seedling establishment (Gendreau *et al.*, 1997).

We sought to determine whether the wave of growth driving the latter stages of Arabidopsis seed germination is also observed in other species, and, in particular, a key crop species. Here we quantify the 3D cell shape changes driving soybean germination in two different positions along the length of the embryonic axis over time.

## Materials and methods

### Germination conditions

Soybean (*Glycine max* L. cv ‘Potência’) seeds were used for all analyses. Seeds were produced under field conditions in Paraná State, Brazil. Seeds were germinated in the light at 22 °C in 90 mm Petri dishes lined with cotton. Germination was scored with the emergence of the radicle.

### Imaging

Samples were prepared using the mPA-PI method as described previously (Truernit *et al.*, 2008; Bassel *et al.*, 2014). Briefly, samples were sliced using a razor and fixed in ethanol:acetic acid 3:1 and 1% (v/v) Tween overnight. Samples were then clarified in 1% SDS (w/v) and 0.2 N NaOH for 1 week. Following rinsing in water, samples were digested by α-amlyase (Sigma Aldrich) overnight in a buffer consisting of 20 mM phosphate pH 7.0, 2 mM NaCl, 0.25 mM Ca_2_Cl. Samples were then placed in 1% periodic acid for 45 min, rinsed in water, and stained with 100 μl Schiff reagent (100 mM sodium metabisulphite and 0.15 N HCl) with propidium iodide added to a final concentration of 100 µg ml^–1^. Following staining, samples were cleared in chloral hydrate solution (4 g of chloral hydrate, 1 ml of glycerol, 2 ml of water). Segments of the soybean axis were imaged using a Zeiss LSM 710 with a ×25 oil immersion lens.

### 3D cell segmentation

Z-stacks were exported as TIFF files in Fiji (Schindelin *et al.*, 2012) and imported into MorphoGrapX (de Reuille *et al.*, 2015) where cells were segmented using a autoseeded watershed following a radial blur of radius 0.5. Segmented stacks were manually corrected for oversegmentation by fusing labels, and meshes were generated using 3D Marching Cubes with a cube size of 0.3 and no smooth passes. The outermost cells whose full 3D shape were not fully captured due to the starting point of the imaging were removed using the clipping planes selection feature to ensure that only accurately captured 3D cell shapes were included in the analysis. Cell layers were selected manually using the mesh selection tool in MorphoGraphX.

### Measuring 3D cell anisotropy and statistical analysis

3D cell anisotropy measurements were obtained by utilizing the protocol for 3DCellAtlas (Montenegro-Johnson *et al.*, 2015). A surface mesh was cast over the sample and trimmed such that only that over the epidermis was retained. A Bezier curve was added through the centre of the steele enabling the alignment of the axes along the principal directions of 3D cell geometry. Individual cell data were exported as CSV (comma-separarted values) for each cell file, position, and time point. Data from four independent samples for each time point [4 HAI (hours after imbibition) and 18 HAI] and each position (H1, H2) were pooled.

Statistical analyses were performed using Mathematica 10.2 (Wolfram Research, Champaign, IL, USA). Violin plots showed that the distribution of each metric was probably non-normal; therefore, 95% confidence intervals for violin plots were estimated using the bootstrapping method, for 1000 iterations each. A correction for multiple comparisons was applied using the Bonferroni correction, for seven comparisons (one per cell layer) following:

$$\text{Corrected interval}=1-\frac{\alpha }{\text{m}}$$

Where *m* is the number of comparisons being made, which yields corrected confidence intervals of 99.3%. This method, while conservative, was chosen for its simplicity.

Ratios were taken by comparing the means of the two sample sets in the comparison, as:

$$\text{Ratio}=e^{\text{ln}\left(\bar{x}\right)-\text{ln}\left(\bar{y}\right)}$$

where $\bar{x}$ and $\bar{y}$ are the means of the two data sets, *x* and *y*, being compared. Confidence intervals were approximated using the following calculations:

$$\text{Interval}=t\left(\frac{S_{ln\left(x\right)}^{2}}{\sqrt{n_{x}}}+\frac{S_{ln\left(\text{y}\right)}^{2}}{\sqrt{n_{y}}}\right)$$

where where $\bar{x}$ and $\bar{y}$ are the two sets of data being compared, *S*^2^ is the variance of a data set, ln(*x*) and ln(*y*) are the natural logarithms of the *x* and *y* data sets, *n*_*x*_ and *n*_*y*_ are the number of samples in each of the *x* and *y* data sets, and *t* is the critical Student’s *t* value for *n*_*x*_+*n*_*y*_–2 degrees of freedom.

Confidence intervals are then defined as:

$$e^{\text{ln}\left(\bar{x}\right)-\text{ln}\left(\bar{y}\right)\;\pm \text{ interval}}$$

### Statistical tests

Student’s *t*-tests were used to compare between time points for each position in a pairwise manner, for each cell layer. The interaction effect (*time position*) of a two-way ANOVA using the model {*time position*, *time*} was used to test for differences in growth dependent on position (growth defined as a difference in a given metric between the two time points). Thresholds for both *t*-tests and ANOVA were adjusted using the Bonferroni correction as per the violin plots for seven comparisons.

## Results

### Sampling soybean axes

**Fig. 1.**
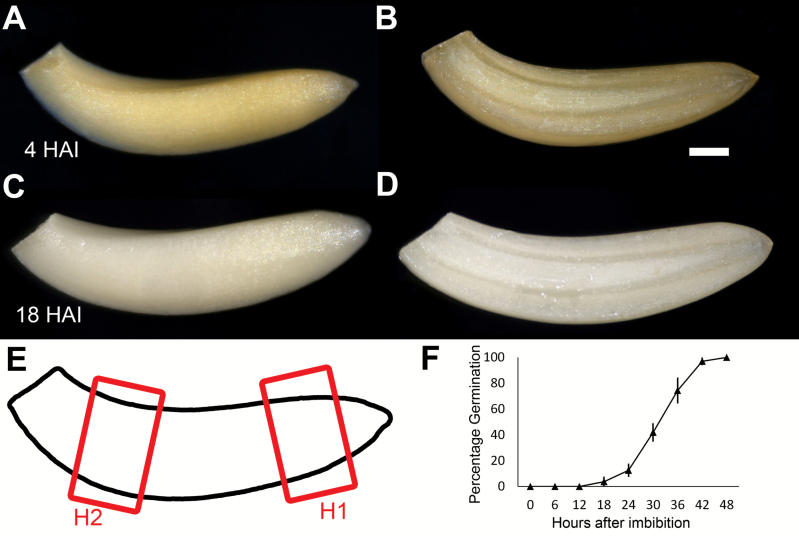
Soybean samples used in this study. (A) Whole soybean axis at 4 HAI. (B) As (A) in longitudinal section. (C) Soybean axis at 18 HAI. (D) As (C) in longitudinal section. (E) Schematic showing the outline of a soybean axis (in black) and the two positions at which the tissue was sampled (H1 and H2), with H1 being closer to the radicle end of the axis. (F) Germination curve of soybean seeds used in this study. Data points are the average of four replicates of 40 seeds. Error bars are the SD.

We sought to examine the differences in the spatiotemporal cell shape changes along the length of the soybean (*Glycine max* L.) embryonic axis during the early germination process. Two time points were selected, one at 4 HAI before cell expansion events began (Fig. 1A, B, F), and another at 18 HAI when a small subset of the population of seeds had begun to complete germination (Figs. 1C, D, F). Individual seeds at 18 HAI that had not completed germination were selected for analysis in order to identify the cell shape changes which occurred immediately prior to the completion of soybean seed germination.

Two different positions along the length of the embryo axis were chosen to look at spatially dependent differences in cell shape change. Each of these areas was selected in domains of the axis which represent the true hypocotyl. The H1 position was in the lower embryo axis, and above the embryo radicle, and the H2 position was in the upper region of the hypocotyl (Fig. 1E).

The whole 3D organ digital capture of cells across the radial width of the hypocotyl (described below) enabled an additional dimension of data to be acquired, namely differences in growth across the numerous cell layers of the soybean hypocotyl.

### Digital capture of 3D cell shape across the soybean axis

**Fig. 2.**
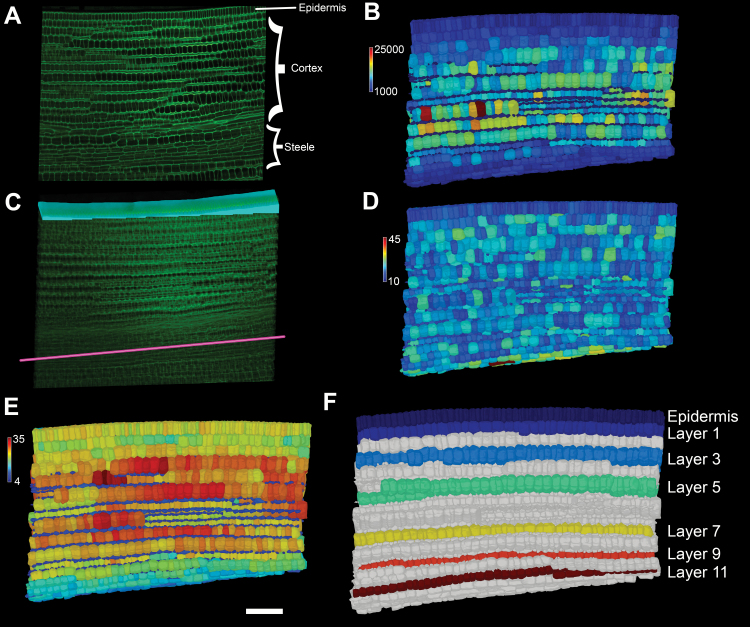
Imaging and computational approach to analysing 3D cell shape changes across the soybean axis. (A) Confocal image section of the soybean axis labelled according to the cell types present. (B) Soybean axis cells segmented in 3D and false coloured for cell volume. The scale bar indicates cell size in µm^3^. (C) Placement of the Bezier curve (pink) and organ surface mesh above the epidermis (cyan) in the confocal stack shown in (A). These guide were used to run 3DCellAtlas. (D) Segmented soybean axis false coloured for longitudinal cell length. The scale bar indicates µm. (E) As (D) but false coloured for radial cell length. (F) Segmented axis false coloured for the different cell layers analysed in this study following manual selection.

To examine the 3D cell shape changes within the different regions of the soybean hypocotyl over time, we performed high-resolution 3D whole-mount imaging using confocal microscopy (Fig. 2A) (Truernit *et al.*, 2008). The process of 3D cell segmentation and meshing was performed using MorphoGraphX (de Reuille *et al.*, 2015), enabling the quantitative analysis of cell size (Fig. 2B).

To calculate the 3D anisotropy of cells within these segmented sections of hypocotyl, we used the 3DCellAtlas computational pipeline (Montenegro-Johnson *et al.*, 2015). Here, a central Bezier spline was manually positioned within the centre of the organ (through the steele), and a surface mesh describing the surface was generated and cropped to cover only the epidermal surface (Fig. 2C). Using the 3DCellAtlas process, three axes representing the principal directions of growth were aligned and used to measure these dimensions within each individual cell (Fig. 2D, E).

In contrast to the Arabidopsis embryo axis which has two cell layers (Montenegro-Johnson *et al.*, 2015), soybean has at least 11 layers of cortical cells (Fig. 2A). In light of the differences in cell organization, we digitally isolated the cells within each of these cell layers to investigate their growth separately throughout this analysis (Fig. 2F). The epidermis, the first cortical cell layer below this, and alternating cortical cell layers thereafter were selected for a total of seven layers of cells (Fig. 2F). Through this process, we also eliminated the small segments representing the air spaces between adjacent cortical cell layers, and inaccurate data given that these segments do not represent real cells (Bassel, 2015). Differences in cell sizes across cell layers were apparent and quantitatively analysed further.

### Analysis of 3D cell shape along the longitudinal and radial dimensions of the soybean axis

**Fig. 3.**
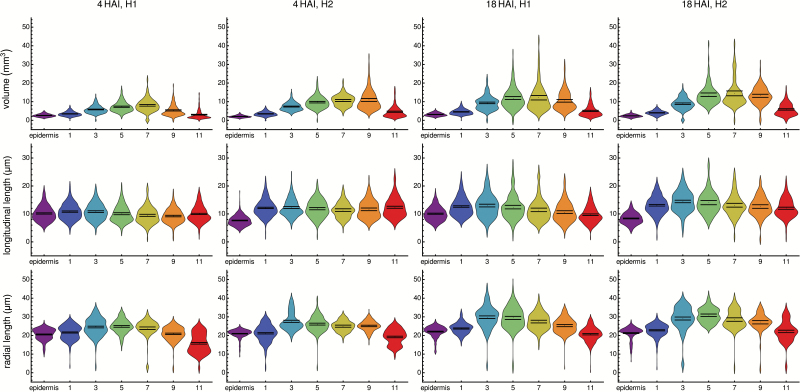
Geometric properties of soybean cells in two positions along the axis and radially within the axis. (A–D) Cell volume in different cell positions in the 4 HAI axis at (A) H1 and (B) H2, and the 18 HAI axis at (C) H1 and (D) H2. (E–H) Longitudinal cell length in different cell positions in the 4 HAI axis at (E) H1 and (F) H2, and the 18 HAI axis at (G) H1 and (H) H2. Radial cell length in different cell positions in the 4 HAI axis at (I) H1 and (J) H2, and the 18 HAI axis at (K) H1 and (L) H2. Cell positions are indicated on the *x*-axis in each plot. Means are indicated with a black dot, and the 95% confidence intervals with horizontal black lines.

Differences in the initial cell size at 4 HAI were observed along the length of the soybean embryo axis in both the H1 and H2 positions (Fig. 3). Cell volume was lower in the epidermis and increased across cortical cell layers spanning the hypocotyl in the radial direction (Fig. 3A, B). Cortical cells in the middle regions of the hypocotyl had the greatest initial cell size, while the innermost cortical cell layer 11 showed a decrease in cell size. This gradient of cell size is in contrast to the Arabidopsis hypocotyl which has two cell layers of roughly equal size (Montenegro-Johnson *et al.*, 2015).

In contrast to cell volume, the initial longitudinal length of cells was relatively uniform across cell layers, with the exception of those of the epidermal layer in the H2 position which were shorter (Fig. 3F).

Radial cell length followed a pattern similar to that of cell volume in both the H1 and H2 positions at 4 HAI (Fig. 3I, J).

At 18 HAI, a time point before the completion of germination, the pattern of cell volume in both the H1 and H2 positions was largely similar to that observed at 4 HAI (Fig. 3C, D). No clear differences in relative cell lengths in the longitudinal or radial directions were observed (Fig. 3G, H, K, L).

### Changes in 3D cell shape along the longitudinal and radial dimensions of the soybean axis

In order to examine the 3D growth of cells at cell layer- and position-specific resolution, we took the data in Fig. 3 and looked at relative cell dimensions between 18 HAI and 4 HAI in each of the H1 and H2 positions (Fig. 1). In light of the differences in initial cell sizes, we focused on changes in relative cell size rather than absolute cell size changes, which is a more accurate indicator of growth (Bassel *et al.*, 2014).

**Fig. 4.**
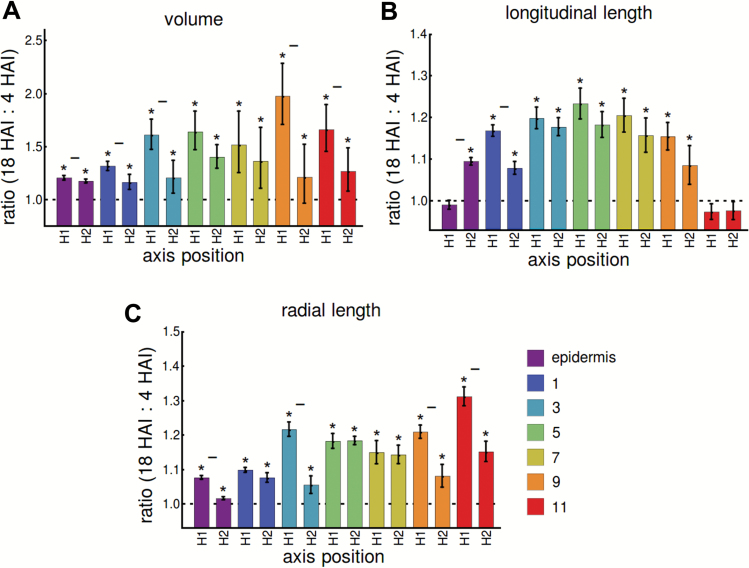
Relative changes in 3D cell anisotropy across cell layers at two positions along the length of the soybean axis during seed germination. (A) Ratio of cell volume between 18 HAI and 4 HAI at different cell positions for each the H1 and H2 positions. (B) As (A) for changes in longitudinal cell length. (C) As (A) for radial cell length. Error bars show 95% confidence intervals for the ratios, asterisks denote significant differences between time points according to a 2-tailed student’s *t*-test, horizontal bars denote significant interaction effects of time and position according to a 2-way ANOVA. All cases are *P* ≤ 0.05, corrected for 7 comparisons.

Cell volume significantly increased in each cell layer at both the H1 and H2 positions between 4 HAI and 18 HAI (Fig. 4A). A greater increase in relative cell volume was observed in the middle cortical cell layers.

Significant differences in cell expansion between the H1 and H2 positions were observed across almost all cell layers, with the H1 position showing greater volumetric expansion than the H2 position. This was true for all cell layers, with the exception of the middle layers 5 and 7 where significant differences were not observed. This observation indicates that there is greater cell growth in the portion of the embryo axis closer to the radicle than the upper hypocotyl up to the final stages of soybean seed germination.

These data also indicated differential growth of cells along the radial direction of the axis which is related to their initial cell size (Fig. 3A). The relative growth contribution of individual cells which have an initially larger size and lower relative contribution by initially smaller cell number is probably a consequence of the spatial constraints imposed by cell size. As a result, more cells can fit into a given space when smaller, and individually their contribution to growth is smaller in relative terms than when there are larger cell sizes. This probably accounts for the differential regulation of cell growth across cortical cell files during soybean germination.

Cell elongation in the longitudinal direction was greater between 4 HAI and 18 HAI in all cell layers, with the exception of cortical cell layer 11 (Fig. 4B). Cell elongation was also greater in the H1 position than in the H2 position in all cell layers, again with the exception of cortical cell layer 11. This indicates that cell elongation is greater in the portion of the embryo axis closer to the radicle than in the upper hypocotyl in most instances. A notable exception to this is cell elongation in the epidermis, where cells in the H1 position grew less than those in the H2 position, and in cortical cell layer 11, where epidermal cells in both positions failed to elongate in length. These unintuitive results suggest that cell elongation in the H1 position principally occurs within the innermost cell layers, while the epidermis and innermost cortical cells are limited in elongation growth.

Growth of cells in the radial direction was significant in all positions between 4 HAI and 18 HAI (Fig. 4C). The pattern of cell anisotropy in this direction contrasted with that of longitudinal growth in several ways (Fig. 4B). These include the greater radial extension of cells in the epidermis and cortical cell layer 11 in the H1 position compared with the H2 position, and diminished differences between H1 and H2 in the middle cortical cell layers, with the middle cortical cell layers 5 and 7 not showing differences in radial growth.

Increases in cell volume in the epidermis and innermost cortical cell layer are therefore primarily accounted for by radial cell elongation, while growth of cortical cell layers 1–7 is principally driven by elongation growth. The expansion of cortical cell layer 9 is a combination of both longitudinal and radial growth.

## Discussion

Here we examined the spatiotemporal patterns of 3D cell expansion and anisotropy during the germination of the crop species soybean. These data collectively point to the greater expansion of cells in the region closer to the radicle than in the upper hypocotyl region of the soybean axis, based on increases in cell volume (Fig. 4A).

This observation is consistent with the previous report of a radicle-derived wave of cell expansion in Arabidopsis (Sliwinska *et al.*, 2009; Bassel *et al.*, 2014). This examination of relative cell growth at two different positions along the soybean embryo axis during germination suggests the conservation in the radicle-derived wave of growth between Arabidopsis and soybean.

The seminal induction of growth-promoting gene expression in Arabidopsis is observed in the radicle, and spreads along the length of the embryo axis during the germination process (Bassel *et al.*, 2014). It has therefore been proposed that the radicle is the cellular site where germination is initiated given that this is the spatial site where the downstream output of the germination programme, induction of growth-promoting gene expression, is first present (Bassel, 2016). The observations presented in this study open up the possibility that the induction of germination in soybean also occurs within the cells of the radicle of soybean given the conservation of this spatiotemporal growth pattern.

Differences in both initial cell size (Fig. 3) and relative growth (Fig. 4) across cell layers were also observed in soybean axes. The non-uniform distribution of cell size across the cortical cell layers of the soybean axis is in contrast to the relatively uniform cell sizes across the two layers of cortex in Arabidopsis (Montenegro-Johnson *et al.*, 2015). Following from this, non-intuitive differences in relative cell growth are observed across the cell layers, probably owing to spatial constraints imposed by cell size differences. The differential growth rates of nearby cells across cell layers suggests the presence of an intercellular feedback mechanism to co-ordinate overall organ growth with divergent cell expansion rates. A mechanical basis for such a mechanism has been proposed previously (Uyttewaal *et al.*, 2012).

Unexpected differences in longitudinal and radial cell anisotropy were also observed in the epidermis and innermost cortical cell layers during soybean germination (Fig. 4B, C). Epidermal cells closer to the radicle failed to elongate, while they did elongate in the upper hypocotyl. This may be related to the forward force being exerted on this region close to the radicle by the elongating axis. Perhaps more surprising, the innermost cortical cells failed to elongate in both the H1 and H2 positions along the axis. While it is not readily apparent why this may happen, intercellular mechanics and regulated cell anisotropy may be playing a role in the control of axis elongation (Hofhuis *et al.*, 2016; Uyttewaal *et al.*, 2012).

Cell layer-specific patterns of cell expansion and 3D cell anisotropy are not intuitive or readily apparent by examining growth on the surface of the organ. Further exploration of gene expression patterns and the mechanical properties of the soybean axis may help explain these complex observations which underlie the germination of this key crop species.
